# Extracts and Abstracts

**Published:** 1889-06

**Authors:** 


### EXTRACTS AND ABSTRACTS.

***A Successful Case of Loreta's Operation on the Stomach.***—By Frederick Treves, F.R.C.S., Surgeon to, and Lecturer on Anatomy at the London Hospital.

So far as I am aware, only one successful case of Loreta's operation for non-malignant stricture of the pylorus has been recorded in this country. This single case was fully reported in the *Journal* for February 19th, 1887. The operator was Mr. Robert Hagyard, of Hull. The patient was a woman, aged 51, who had been troubled with gastric symptoms for five years. She had become greatly emaciated; she was unable to take any food by the mouth, and the stomach was extremely dilated. No tumor could be detected in the vicinity of the pylorus. The operation was performed on March 7th, 1886, and the woman made an uninterrupted recovery. Mr. Hagyard, in a letter to me dated February 21st, 1888, writes: "The patient is perfectly well, there being no return of the symptoms of obstruction. The dilated stomach, which was so difficult to lessen, has now subsided, and my patient has just recently married again." A more admirable result modern abdominal surgery could scarcely claim.

The present patient was a carman, aged 27, who was admitted into the London Hospital under the care of Dr. Ralfe, on October 12th, 1887. He was suffering from vomiting and from severe gastric pain. There was nothing noteworthy in the patient's family history. With regard to himself, he had never had any previous illness of a definite kind. He had, however, led an irregular life, and had been a heavy drinker. His general health had suffered in consequence of his intemperance. He had never had syphilis. Three years ago he was kicked by a horse in the epigastrium. He became collapsed, and suffered much abdominal pain; he believes he vomited. He thought little of the injury, and was only laid up seven days. Eleven months before his admission into the hospital he began to experience gnawing pains in the belly in the region of the epigastrium. Previous to this he had had attacks of "disordered stomach," had impaired appetite, and occasional "bouts" of vomiting. He ascribes these symptoms to excessive drinking. The gnawing pain was a new feature. The pain increased in severity and in duration. It was paroxysmal, came on at intervals of one to three days, and appeared usually a short time after a meal. Very soon each attack of pain was followed by vomiting. The matter ejected was described as brownish, and he states that on each occasion he brought up a quart or so. The vomiting gave him relief.

The pain became more severe, and shot through to his back. He never passed a day without vomiting; he began to lose flesh, and was afraid to take food. During some of the attacks he stated that he became a little yellow. He does not appear to have ever had distinct jaundice. Some months before admission he had commenced the use of morphine, which he ultimately took on every opportunity. He now suffered from constipation.

On admission (October 12th, 1887) he was very feeble, anæmic, and much emaciated. He complained of an incessant gnawing pain in the right hypochondrium, and of occasional attacks of severe paroxysmal pain in the same region. During these attacks he would roll about in bed and scream, but it is possible that the paroxysms were a little colored by his craving for morphine. The pain came on every few hours on some days, and was described as resembling colic. It was almost immediately induced by taking food. So marked was this that he was afraid to eat. He vomited copiously at least once every day, and was troubled with eructations of foul gas. The bowels acted about once a week. The tongue was broad, pale and flabby, the pulse feeble, the temperature normal. An examination of the abdomen revealed a great dilatation of the stomach; there was much tenderness complained of in the epigastric and right hypochondriac regions. The seat of the greatest pain was a spot about one inch and a half above the umbilicus. This pain was always described as shooting back to the spine. There was no jaundice; the urine was scanty but normal; the liver, spleen, lungs, and heart were reported as healthy. On the day after admission the patient vomited three quarts. When the stomach was empty, a little dullness was made out in the vicinity of the pylorus. It blended with the area of hepatic dullness. No tumor of any kind could be detected. The patient had never vomited blood, nor does blood appear to have been passed by the bowel.

The stomach was washed out daily, and very little food was given by the mouth; nutrient enemata were commenced, and the pain was a little modified first by salicylic acid and later by cocaine. Although the pain and the vomiting were lessened, the patient did not exhibit any general improvement. He continued to waste and to lose strength.

On October 31st, œdema of both feet appeared.

On November 14th the report was to the effect that he was more emaciated and more anæmic, and was so weak as to be unable to sit up in bed. He was now fed solely by nutrient enemata, taking only a little fluid by the mouth. He was always found lying on the left side, with the knees drawn up.

On November 30th it was evident that the patient could not live many days longer unless he was relieved. To his previous symptoms diarrhœa had been added. He was extremely feeble and utterly apathetic.

Dr. Ralfe had formed an opinion that the case was one of non-malignant stricture of the pylorus. At his request, I arranged to make an exploratory incision, and if necessary to dilate the pylorus.

The operation was performed on December 3d, 1887. Chloroform was administered, and the operation was carried out under the spray. Carbolic acid lotion of the strength of 1 in 40, and warmed to the temperature of 90° F., was used throughout the operation. All the sponges used were warmed.

I made a vertical incision, four inches in length, in the median line. The lower end of the cut reached as far as the umbilicus. The greatly dilated stomach was at once exposed. The pylorus was at first difficult to define. It appeared to be imbedded in a mass of almost cartilaginous hardness, which was firmly adherent to the under surface of the liver. Not only was the actual pyloric extremity of the stomach adherent to the liver, but a portion of the viscus itself, to the extent of some three square inches, was in like manner attached.

It was evident that the adhesions were inflammatory, and that they were especially tough and thick in the region of the pylorus. I divided the adhesions as freely as was possible. The segment of the liver to which they were attached was pale, and had the appearance of being atrophied. It was found impossible to quite free the pylorus, and to entirely separate the stomach from its attachment to the liver.

I then made a vertical opening into the stomach midway between the two curvatures and about two inches from the pyloric orifice. Before this opening was made the stomach was drawn as far forward into the wound as possible, and was gently held by a small pair of dressing forceps, the blades of which were protected by india-rubber. The interval between the viscus and the parietes was plugged with sponges so that any fluid escaping from the stomach would have been prevented from reaching the peritoneal cavity. To avoid bleeding in opening the stomach I think that the incision should be vertical, and should not be too near the pylorus. It should be scarcely large enough to admit the index finger, with which it is at once plugged. In Mr. Hagyard's case, the bleeding at this stage of the operation is described as "terrific." A rush of escaping gas followed the opening of the stomach, but as the wound was at once plugged with the right forefinger no fluid escaped and there was no bleeding. Within the stomach the portion of the gastric wall still adherent to the liver could be well made out. It was perfectly smooth. The pylorus was surrounded by a ring of very dense tissue, and appeared to be set in cartilage. It would not take the point of the forefinger. I steadied the pylorus with the left hand while I gradually bored the right forefinger into the constricted orifice. The process of dilatation was slow, but in time the finger was introduced into the duodenum. Without withdrawing the finger from the stomach I enlarged the wound in the viscus with a bistoury held in the left hand, and passed the middle finger into the stomach. The wound even now was small, and the fingers were closely embraced by the gastric wall. Under such conditions hæmorrhage was scarcely possible. The middle finger was passed through the pylorus, and the forefinger inserted slowly after it. In a little while the orifice was sufficiently dilated to take the two fingers. During the process the pylorus was steadied by the left hand. This dilatation appeared to me to be quite sufficient, because an orifice admitting the two fingers together would have a circumference of about four inches. Loreta is reported to dilate the pylorus with the two forefingers until they are more than three inches apart. This would represent an opening with a circumference of not less than seven inches. Had dilatation to this extent been attempted in the present instance the walls of the viscus would certainly have been ruptured.

The wound in the stomach was now closed by a continuous suture of fine silk. This suture included the mucous and muscular coats, and was introduced by means of a Hagedorn's needle. The silk was so applied as to arrest some little bleeding that was occurring from the edges of the incision. The ends of the silk were cut short, and the wound was then completely closed by many points of Lembert's suture, which involved the serous coat only The parts around the operation area having been cleansed, the abdominal wound was closed by deep interrupted sutures in the usual way. The wound was dressed with a single sponge dusted with iodoform. This was firmly secured in position by means of a broad and tightly applied flannel binder. The temperature on the evenings of the third and of the eleventh days rose to 100°; with these exceptions it remained normal.

The patient at once experienced relief from the operation. He was kept under the influence of morphine, and was fed by nutrient enemata; he was allowed to suck a little ice to relieve thirst; there was no return of the vomiting. On the seventh day the stitches were removed, and the wound supported by gridiron strapping. On the evening of this day the patient vomited a little; this was the first and only time that he was sick after the operation. He became greatly excited during this attack of vomiting, sat up in spite of all remonstrances, and made violent efforts to get out of bed; he persisted in yelling for nearly an hour. As a result of these exertions the wound, which had healed by first intention, gave way, and the stomach was once more exposed. The margins of the incision were at once brought together by means of long strips of plaster, and a strong binder was applied. As the sickness was apparently due to the decomposition of accumulated mucus in the dilated stomach, that viscus was now washed out every other day with a little warm boracic lotion. This gave the patient great relief; the nutrient enemata were continued.

On the ninth day a little milk and soda water was given by the mouth; the quantity of food thus administered was gradually increased each day. The nutrient enemata were continued until the fourteenth day, when they were finally omitted. The patient after that date took all his food by the mouth.

On the eleventh day some solid food was given in the form of toast soaked in tea. The washing out of the stomach was discontinued on the seventeenth day. The patient complained of no pain, but remained very restless, and persisted, except when restrained, in sitting up in bed. The wound healed by second intention. The bowels acted regularly and without aid after the enemata had ceased to be used.

On the 20th day the patient was allowed to leave his bed. Four days after this he insisted upon leaving the hospital, and walked without assistance to the door. He had now no pain and no sickness and was taking his food well; the stomach was, however, still much dilated, and the wound was not firm. It was intended that massage should be applied to the abdomen, but no arguments would induce the patient to remain in the wards.

The man presented himself at the hospital at the end of two or three months. He was greatly improved in health, could eat well, was never troubled with sickness, and had no difficulty with his bowels. He complained of a sense of weakness at the wound. The cicatrix had yielded considerably, leading to some protrusion of the contents. A stout belt was ordered, and with this he expressed himself as feeling quite comfortable.

I saw the patient again in December, 1888, about twelve months after the operation. He now complained only of poverty. He had an excellent appetite, and was troubled with neither sickness nor dyspepsia. He had returned, so far as his very limited means would allow, to his previous intemperate habits. He still wore his belt, and the stomach appeared to have entirely returned to its normal dimensions.

It is evident that this operation must of necessity be of very limited application, since the examples of non-cancerous stenosis of the pylorus are quite rare. The procedure has been carried out with astonishing frequency on the Continent, and it would be interesting to know the precise pathological condition which has been found to be associated with these operations. The specimens in the various museums in London throw little light upon the fibrous stricture of the pylorus of which so frequent mention is made in Continental literature.

In the present case the narrowing of the pylorus appeared to be, to a great extent, due to the contraction of peritoneal adhesions. The trouble may have originated in an injury to the stomach due to the kick from a horse, or may have followed upon a perforating ulcer at the pyloric extremity of the viscus.

---

***Epithelioma of the Lip, Based on the Records of 100 Cases.***—By W. Roger Williams, F.R.C.S.

Analytical Summary of 100 Cases of Epithelioma of the Lower Lip in Males.

Seventy-two of these cases were consecutively under treatment at the Middlesex Hospital during the last 13 years, the other 28 cases were under treatment at University College Hospital during the 7 years 1880-86, *q.v.* the Registrar's report.

Age.—The earliest age at which the disease was *first noticed* was 26 years, the latest 75.5 years, the mean age 52.4 years.

The numbers for each quinquennial period in 100 cases are as follows:—

| Age. | Cases. | Age. | Cases |
|---|---|---|---|
| 25 to 30 years | 5 | 55 to 60 years | 13 |
| 30 to 35 " | 3 | 60 to 65 " | 14 |
| 35 to 40 " | 7 | 65 to 70 " | 9 |
| 40 to 45 " | 10 | 70 to 75 " | 4 |
| 45 to 50 " | 14 | 75 to 80 " | 1 |
| 50 to 55 " | 20 | | |

Total Duration of Life.—In 19 fatal cases the total duration of life, dating from the time when the disease was *first noticed*, averaged 37.7 months.

(*a*) In 7 of these cases the disease ran its course *without any operative interference;* their average duration of life was 16 months, the longest 31, the shortest 7 months.

(*b*) In the other 12 cases the *primary disease had been excised;* their average duration of life was 50.5 months, the longest 96.6, the shortest 10 months.

Duration of Life Subsequent to Excision of the Primary Disease.—In 12 cases the average was 29 months, the longest 96, the shortest a few hours. The subject is further illustrated by the subjoined table:—

*Duration of life subsequent to excision of primary disease in 12 cases.*

| Age. | Cases. | Age. | Cases. |
|---|---|---|---|
| Under 1 month | 1 | 24 to 30 months | 2 |
| 6 to 12 months | 2 | 48 to 54 " | 1 |
| 12 to 18 " | 2 | 60 to 66 " | 1 |
| 18 to 24 " | 2 | Over 66 " | 1 |

The Original Seat of the Disease.—This was noted in 59 cases, with the following result:—

| | |
|---|---|
| R. half of lower lip in | 21 cases. |
| L. half of lower lip | 21 " |
| Middle of lower lip | 13 " |
| Angle of mouth (L.) | 4 " |

The Mode of Origin.—In 30 cases the initial lesion is described as having presented the following characters:—

| | |
|---|---|
| Small sore or ulcer in | 16 cases. |
| Small hard nodule or lump | 7 " |
| Crack | 5 " |
| Hard white speck | 1 case. |
| Wart | 1 " |

Out of 50 primary cases, more than a single cancerous lesion presented in five, but in every case the disease was unilocal in its origin.

Occupation.—This was stated in 36 cases as follows:—Farm laborer in 5 cases, general laborer in 5, sailor in 3, bricklayer in 2, and 1 each of the following: saddler, cowman, blacksmith, stoker, paper factory, piano factory, sewerman, bailiff, gardener, brazier, carpenter, gasfitter, costermonger, coachman, carman, commercial agent, boatman, waiter, soldier, foreman, and groom.

Ætiological.—There was a history of previous injury or disease of the lip in 35% of all the cases.

Of 29 cases in which inquiries were made there was history of previous *injury* in 6, irritation by rough tooth in 1, by plate of artificial teeth in 1, by scratch from meat hook in 1, by holding copper nails in lips in 1, by sting of wasp in 1, and by blow from a stone in 1 case.

Of 26 cases there was history of previous *disease* of the lip (ichthyosis) in 3.

Of 26 cases in which inquiries were made as to *smoking*, etc., 16 had been great smokers, and 8 moderate smokers; 5 of the smokers had chewed as well; 2 had never smoked nor chewed.

Previous Health.—Of 25 cases in which inquiries were made, the previous health is stated to have been good in 24 (with no serious illness since youth in 8), and in 1 case it had been indifferent. Syphilis is *not* included in this statement.

*The occurrence of Cancer.*—Of 87 cases in which this point was specially investigated, there was history of *cancer* in 5 families, or in 5.7 per cent. The *relatives* thus affected and the *seats* of the disease were as follows:—

1. Patient's 2 sisters died of cancer, 1 of *breast* the other of *uterus.*
2. Patient's *mother* died of cancer of *breast.*
3. Patient's *father* died of cancer of *cheek*, and his *mother* died of "internal cancer."
4. Patient's *father* died of cancer of *lip.*

Primary or Recurrent.—73 cases were primary and 46 recurrent. Several of the primary cases manifested recurrence before leaving hospital, and are here reckoned twice.

The average *interval between the first operation* and the *first obvious recurrence*, in 41 cases, was 20 months, the maximum 110 months.

The following table further illustrates this subject:—

*Intervals in monthly periods. Numbers for each period in 41 recurrent cases.*

| Age. | Cases. | Age. | Cases. |
|---|---|---|---|
| Under 1 month | 2 | 24 to 30 months | 5 |
| 1 to 2 months | 3 | 36 to 42 " | 2 |
| 2 to 3 " | 6 | 48 to 54 " | 2 |
| 3 to 4 " | 4 | 60 to 66 " | 2 |
| 4 to 5 " | 3 | 66 to 72 " | 1 |
| 5 to 6 " | 2 | 72 to 78 " | 1 |
| 12 to 18 " | 7 | Over 110 " | 1 |

In 29 of these 46 cases the initial situation of the recurrent disease was at the *primary seat*, in 4 cases it was *near the primary seat*, but not at it, the scar of the former operation being quite sound.

In the other 13 cases the initial situation of the recurrent disease was in the *adjacent lymphatic glands*, the scar at the seat of the primary disease remaining healthy.

*Condition of the lymphatic glands.*—In 72 *primary* cases there was obvious enlargement of the adjacent lymphatic glands in 36, or in 50 per cent.

In 28 recurrent cases there was obvious glandular enlargement in 25, or in 89 per cent.

Treatment and Result.—The disease was *excised* in 64 *primary* cases with but a single death.

In 51 cases the *lymphatic glands were not touched.* All of these recovered. The average duration of their stay in hospital was 18 days.

In the other 13 cases *glands were dissected* out as well. The single fatal case belongs to this group. This patient died of traumatic pneumonia shortly after prolonged artificial respiration for syncope under chloroform. The average period of their convalescence 20 days.

There were 20 operations for *recurrent* disease, without a single death. In 11 of these cases *glands were dissected* out. The average duration of their stay in hospital was 46 days. The average period of convalescence of the other 9 cases, in which *no glands were removed*, was 15 days.

The *causes of death* in 12 other cases were as follows: Asthenia in 8, œdema of glottis in 2, hæmorrhage in 1, and cystitis with suppurative nephritis in 1.

Lymphatic Glands.—In every case but one the adjacent lymphatic glands were infiltrated; in 8 cases those of *both* sides of neck and the submental; in 3 cases those of left side of neck; and in 1 case those of right side.

In 6 cases the *inferior maxilla* was infiltrated and atrophied, and in 4 necrosed as well.

Secondary Deposits.—Of these 13 necropsies there were secondary deposits in other parts of the body in 2 cases, or in 15.4 per cent.

The parts thus affected were:—

Lungs (R. 1, L. 1) in.........2 cases.
Kidney (R.).................1 case.

—*The Medical Press*, May 1, 1889.

---

***Peculiar Action of Potassium Iodide in a Case of Syphilitic Rhinitis.***—Reported by Frank P. Hudnut, M.D., to the Medical Society of the County of Kings, February 19th, 1889.

Mr. D——, aged forty-five, a widower, of sober and temperate habits. Four years ago he contracted syphilis, which ran the usual course. For the past six months he has had more or less discharge from the nose with considerable pain in the frontal region. The discharge was of a greenish color and very fetid at times.

Upon examination, I found a condition of syphilitic rhinitis. The septum was swollen and ulcerated, about an inch back from the meatus. Upon the introduction of a probe, I discovered necrosed bone, and passed the probe into the other nostril through a small opening.

Spraying the parts well with cocaine, I removed, with a dressing forceps, several large pieces of necrosed bone, amounting in the aggregate to the size of a two-cent piece. Upon examining the larynx, found the parts normal, cleansing the nose thoroughly with permanganate of potash, two grains to the ounce, and coating the membrane with iodoform, I exhibited ten grains of the iodide of potash three times a day, and requested him to call again in three days. At subsequent visits I removed more bone, until healthy tissue was reached, but not until nearly all the septum had been removed, the cartilaginous portion remaining intact.

As there continued to be a large amount of matter discharged from several ulcerated spots, I made an application of nitrate of silver, fused on a probe, to these spots, and sprayed the nose with a ten grain to the ounce solution of argenti nitras. After using this solution several times the discharge ceased. As there was considerable hypertrophy of the turbinated bones and some deflection of the septum (cartilaginous portion), I removed the superabundant parts with the cautery and knife, being careful not to weaken the septum too much. I had been increasing the iodide every visit until he was taking ninety grains a day up to this time (about three weeks).

No symptoms of iodism were observed, unless it was a slight increase in the action of the bowels and an appreciable metallic taste. His general health improved very much, and he expressed himself as feeling very well. After he had been taking the ninety grains of iodide each day for three days, he came to me very much alarmed. I found he had an attack of subacute laryngitis, his voice was very hoarse, and his larynx very much inflamed. After cleansing the parts well with Dobell's solution, I sprayed the larynx with argenti nitras, 20 grains to the ounce. Continuing this treatment for several days and finding no improvement, I decided to stop the iodide and see what effect this would have. On going three days without the iodide, I noticed a decided improvement in his condition. Being aware of the fact that iodide would produce coryza, and in some cases a a slight laryngitis, I became very anxious to find out if the iodide was producing the irritation. In order to decide this, I directed him to resume the iodide again in the same doses as before, and to report in three days. In the meantime I gave him no treatment.

Upon the next visit I found him even worse than at the beginning of his trouble; he could not speak above a whisper. I then stopped the iodide entirely, giving no treatment. In three days he was much better, and at the end of a week he was well. I then reduced the dose to ten grains three times a day, and since then he has had no trouble.

In looking up the effects of iodide on the larynx by various authors, only slight reference is made to the action of the iodide on the larynx, and then only when iodism has been produced. Bartholow describes it as a hoarseness and accompanied by coryza, with a rise of temperature. I cannot but feel in this instance that the inflammation of the larynx was entirely due to the iodide.

My object in making note of this case was to draw your attention to the liability of throat complications when using iodide in the treatment of syphilitic affections. Of course the danger is very slight and you might treat many cases without finding one presenting similar symptoms; still, it is well to bear in mind the danger of such complications.—*The Brooklyn Medical Journal.*

---

***Enteric Fever in India.***—Having recently discussed this subject, we only revert to it now because our attention has been invited to the letters of "Medicus," published in the *Pioneer*, one of the leading newspapers in India. "Medicus," while of the opinion that the earth system of disposing of sewage is the best for India, if carried out in a proper way, shows that in certain important cantonments this is very far from being the case. Take the following description: "The trenches are dug often two or three feet deep, and are more than half filled with sewage, then covered over with refuse. In the dry weather these trenches rise into mounds, and in the rains sink into quagmires of decomposing filth, becoming the very breeding grounds of enteric fever." "Medicus" dwells on what he conceives to be a mistake on the part of the authorities. Great expense has been incurred, and properly incurred, in bringing a supply of as pure water as is attainable into cantonments, but leaving the bazaars, in the close vicinity of the military stations, unsupplied with the same. The result being, as has been a thousand times pointed out, that the aerated drinks which the soldiers consume in such places are made with water grossly impure. Another source by which the enteric fever is carried into the system is by the underclothing of the men being washed in tanks which are nothing else than imperfectly diluted sewage. The same author presses a point which is worthy the serious attention of the authorities in the face of the yearly increasing mortality from enteric fever.

At present the sanitation of our cantonments is chiefly in the hands of cantonment magistrates, a body of officers quite unfit for the work of health-officers. At best they have zeal without knowledge; many of them are without either. Thus it comes about that sources of disease are left uncared for, often, as we have just shown, from ignorance, as often from indifference. There is some evidence in the letters under notice of a danger growing up near our cantonments in India strictly analogous to one that was a powerful factor in the propagation of yellow fever in the West Indies. Sewage is, no doubt, removed from the immediate vicinity of the barracks, but it is too often disposed of in the manner thus described by "Medicus:"—

"A field of a few acres is taken some distance from the barracks. A track, not a regular road, leads to it; by this the sewage, solid and fluid, is carted. Some half a dozen sweepers at most dig trenches about ten feet long by one foot wide and two or more in depth; into each trench one cartload or more of filth is placed, which is then closed with loose earth. The site is subsequently cultivated or not; in many cases it is not, but in either case the amount of filth is so great and at such a depth that no cultivation can purify it; in fact, it is hidden and not purified. If the spores of glanders, after the cremation of animals dying from that disease, exist in the soil where this *débris* has been buried after a laspe of twenty years, what under the above conditions can we expect of those of enteric fever? They require heat and moisture for their development, and under this system of sanitation they are given the very conditions necessary to their production. In every large cantonment at least 500 acres should be devoted to a sewage farm; it should be carefully fenced in. The excreta should be treated with dry earth, to which a small quantity of lime might be added when placed in the receptacles before being carted off. The resulting compound should be placed on the surface of the ground and dug into the soil, trenches never being used. This ground ought not to have a fresh application for five years."

"Medicus" is of opinion that the only remedy for this dangerous condition of things is the appointment at the great centers of military population in India of health officers. This means money; alas! there is, there can be, no efficient sanitation anywhere without the expenditure of money. Our author seems to think that some retrenchment for this purpose could be made in the direction of the great amount spent in the "higher education" of a certain class of natives, who, we must admit, seem disposed to turn this "education" to a bad account. It should, at all events, be kept in mind that every European soldier whose life is wasted is a money loss to the State of £200.—*The British Medical Journal*, April 27, 1889.

---

***Notes of a Case of Sclerema Neonatorum.***—Reported by Alfred G. Barrs, M. D. On March 6th, 1888, A. Y., aged one month, apparently in good health, was brought to me for an intense livid redness of the nates, corresponding pretty much to the surface covered by her napkin, and extending downwards well into the popliteal spaces. The surface, which was uniformly affected, was dry and polished, and the redness disappeared to a great extent on pressure. The appearances were obviously not due to any eczematous condition. The lower limits of the condition tailed off into papules of varying size, which had not the same characters as the general body of the disease. Looked at more closely, the change was seen to affect the parts limited above by the iliac crests; below by the lower borders of the popliteal spaces, and laterally by the line of the ilio-tibial band. Over the whole of this area the skin and subcutaneous tissue, and it almost seemed the still deeper parts, felt as though infiltrated and welded together by some solid material, producing a very close resemblance both in color and consistency to that of an uncooked ham. The firmest pressure produced not the slightest pitting. The pudenda were not affected. The cleft of the nates had been reduced to a mere line by the mutual pressure of the two sides, converting its naturally rounded surface into a sharp rectangle. It was quite impossible to separate the skin from the subjacent parts at any point in the affected area. One or two isolated patches of a precisely similar nature were found in the right deltoid region. There was nothing in the family history, or in the child's condition, to suggest syphilis or any diathetic or local cause for the disorder. The mother stated that the condition had been first noticed on the fourth day after birth (though it might have been earlier) on the back of each thigh, just above the popliteal space, and had gradually spread till it had attained the limits above described in the course of about ten days, since which time it had been stationary or nearly so. I ordered the parts to be protected as much as possible from any excoriation by the napkin and excretions, and half a grain of grey powder to be administered night and morning.

On March 13th, the redness was a little less marked, the surface was still dry and polished, and except that there was only a slight disappearance of the condition at its lower part, the brawny state continued the same in character and distribution. The child's general condition continued to be quite good.

On March 20th, the child was growing and thriving, and was clearly not in the least affected by the local condition. The hardness was disappearing, its margins retiring towards the centers of the buttocks. The redness was much less.

On April 3rd the condition had almost entirely disappeared, and on May 1st the child was reported to be quite well in every way.—*The British Medical Journal*, May 4th, 1889.

---

*The Treatment of Ingrowing Toenail.*—As I have had some very troublesome cases of the above to treat, I may be allowed, perhaps, to supplement Mr. Scott Battams' memorandum by advice which will, I trust, not only aid in the cure, but, what is far better, prevent the occurrence of this troublesome complaint, and when cured, prevent its recurrence.

In the first place, I never remember seeing a case where the inner side of the great toe was affected. As a rule, it is the outer side. I believe the complaint is due to pressure of the great toe on and against the second toe, by narrow-toed boots; this naturally has a tendency to push the flesh over the outer angle of the nail.

In the second place, I am inclined to think the complaint is caused, and certainly it is aggravated, by cutting the nail down at the corners, and not allowing it to grow square; this, *plus* the pressure of the boot, may account for the inner side of the toe becoming affected.

Recognizing these as the two main causes of the complaint, the preventive treatment is obvious, namely, allowing the nails to grow square, never to cut down at the corners, and the wearing of boots sufficiently wide and deep to prevent any pressure on the toes. For the cure of the complaint I would recommend an elongated wedge of cotton-wool, lint, or soft linen rag (as more likely to be retained than sponge) carefully tucked in (with a wooden match, flattened at one end) between the flesh and the nail, the end of the wedge being tucked under the angle, as far as possible, of the offending nail, which may sometimes be found buried away under the granulations. The relief at once is great, and a complete cure usually results in a week or so.

The nitrate of silver or other caustic may sometimes hasten the cure, but I have never found it necessary to use strapping over the cotton-wool, as this must cause some pressure when walking, and, if a proper quantity of wool be tucked in firmly but gently, it will be retained till the following day, when it should be changed.—*G. F. Poynder, in The British Medical Journal.*

---

***Case of Salivary Calculus: Removal.***—Francis W. Clark, L.R.C.P., reports in the *British Medical Journal* the following case which is of interest from the comparative rarity of cases of salivary calculus, and also from the long duration of the symptoms.

J. B., a young man, aged 29, applied to me on the morning of Tuesday, February 12th, complaining of a swelling under the left angle of the lower jaw. This tumor, on first examination, was found to be about the size of a horse bean, and the patient stated that every time he ate a meal, or even smelt a savory dish, the tumor rapidly swelled, and sometimes attained the size of his fist, in the course of a few minutes. When swollen the tumor caused him considerable pain; but as it subsided, which it did in the course of some half hour or less, the pain also ceased. He stated that he had had the swelling for more than four years, and had, on several occasions, been advised to have the tumor removed, but had demurred.

My first impulse was to test the man's statement as to the tumor swelling up so rapidly, and accordingly I gave him an apple to eat, and I found that the swelling did rapidly increase to several times its former size. I next examined his mouth, and found that the cause of all his trouble was a salivary calculus, blocking Wharton's duct, and I then elicited from the patient that ever since he had noticed the swelling he had been troubled with dryness of the mouth, which was most noticeable on the left side. I explained the circumstances of the case to the patient, and he at once agreed to my removing the calculus; but I found that it was impossible to do this single-handed, owing to the difficulty in fixing the tongue, so I then endeavored, and was fortunately successful, in opening Wharton's duct between the calculus and the gland, and so obviated any further swelling of the gland by the accumulation of the saliva. I then arranged that he should come to me again on the following Sunday, and on that day I was able, with the valuable assistance of my friend, Mr. H. Loyd-Williams, to remove the calculus. The patient informed me on this day that the gland had not swelled up at all since my former operation, but he complained somewhat of an excess of saliva in the mouth, and of an unpleasant mawkish taste. This was due, no doubt, to his having been for so long accustomed to a comparatively dry mouth, and the taste to the discharge into the mouth of saliva, which had long stagnated in the gland.—*Brit. Med. Jour.*

---

***On the Cocaine Habit in Diseases of Throat and Nose.*** Lennox Browne, in the *British Medical Journal.*—From the frequency with which samples of so-called voice lozenges containing cocaine are forwarded to me by druggists, I presume there is a demand for the same, unless, indeed, as is not unusual in the present day, the pharmacist is endeavoring to educate the physician into the conviction of a want which he had not hitherto experienced. I also observe that patients are frequently prescribed, or obtain without prescription, cocaine solutions of considerable potency, for constant use by brush or spray with the purpose of relieving quite slight symptoms of chronic conditions. I venture, therefore, to utter a word of warning against this growing inclination to cultivate a cocaine habit; and without entering into the question of constitutional evils likely to accrue from such a practice, I feel bound to emphasize the injurious local effects on the naso-pharyngeal and laryngeal mucous membrane which are certain to result from frequent application thereto, whether by means of brush, spray, insufflation, or lozenge, of a drug which produces topical anæsthesia by an emptying of the surface capillaries.

Some remarks which I made of a similar intent in the Section of Laryngology at the International Medical Congress, held at Washington in 1887, were received with general approval by the many eminent specialists who were then present, and I feel confident that this short note will be endorsed by the majority of my co-laryngologists in this country. * * *

---

***Catalepsy in Mother and Child.***—A case was recently reported in the *Nederlansch Tijdschrift voor Geneeskunde*, where a pregnant woman became cataleptic, her child suffering from the same affection. The patient was a robust woman, aged 44; she had borne eleven children. In youth she suffered from typhus, and after recovery became subject to fainting fits, but throughout her married life she remained strong and well. There was no history of any neurosis in the family. In the seventh month of her twelfth pregnancy she was seized with cataleptic fits, following the loss of a child. Dr. Schoot, of Leeuwarden, found her stiff and motionless. The forearm could be raised and bent with some force, and remained in the same position for about ten minutes, then it slowly fell. The lower extremities could be treated in the same manner, with the same effect. Consciousness was lost. The pulse was 64, full and regular; the temperature normal; the respirations natural. The pupils, somewhat dilated, reacted to light. On inhaling chloroform the rigidity of the muscles disappeared, and the patient seemed to sleep calmly for four hours. On awaking from the fit the patient remembered nothing that had taken place while it lasted. The fœtal heart sounds, previously audible, were lost, and not heard till fourteen days before labor. No albumen could be found in the urine. The cataleptic fits occurred about three or four times daily, sometimes there was an interval of two days. Atropine gave the patient a week's repose. The fits continued to term, when she was safely delivered of an apparently healthy boy. Not for four days later did the fits recur. On the fifth day an attack occurred while she was suckling; two days later another fit took place, but no more came on, and three months after labor she was quite well. Shortly after the first attack after labor the child, who had been weaned because of that attack, was seized with dysphagia, and could hardly swallow cow's milk. In the evening it had a cataleptic fit. The symptoms were precisely the same as in the mother. The flexibilitas cerea of the limbs was marked. The muscles relaxed during a warm bath, but soon afterwards tonic cataleptic convulsions occurred, and the child died after two days' duration of the fits. Dr. Schoot, after due consideration of the differential diagnosis of the case from eclampsia and hystero-epilepsy, came to the conclusion that the fits were cataleptic both in the mother and the child. That they were identical in each case there could be no doubt, but the precise relation of the affection in mother and child remained obscure.—*The British Medical Journal*, May 11, 1889.

